# Transvaginal Strain Elastosonography in the Differential Diagnosis of Rectal Endometriosis: Some Potentials and Limits

**DOI:** 10.3390/diagnostics11010099

**Published:** 2021-01-09

**Authors:** Marco Scioscia, Antonio Simone Laganà, Giuseppe Caringella, Stefano Guerriero

**Affiliations:** 1 Unit of Gynecological Surgery, Mater Dei Hospital, 70125 Bari, BA, Italy; 2 Obstetrics and Gynecology, Mater Dei Hospital, 70125 Bari, BA, Italy; caringellagiuseppe@libero.it; 3 Department of Obstetrics and Gynecology, “Filippo Del Ponte” Hospital, University of Insubria, 21100 Varese, VA, Italy; antoniosimone.lagana@uninsubria.it; 4 Obstetrics and Gynecology, University of Cagliari, 09124 Cagliari, CA, Italy; gineca.sguerriero@tiscali.it; 5 Department of Obstetrics and Gynecology, Azienda Ospedaliero Universitaria, Policlinico Universitario Duilio Casula, 09045 Monserrato, CA, Italy

## Dear Editor,

We sincerely thank Szabó et al. [1] for their comments on our article [2] and their proposal for improving the differential diagnosis of bowel endometriosis by transvaginal elastosonography (ESG). This technique provides noninvasive information on the elasticity and stiffness of a lesion, as it is based on the principle that the compression of soft tissues produces a greater strain in soft and elastic lesions than in harder, more rigid lesions. The results (calculated strain ratio) depend on the amount of the fibrotic component of the lesion and the surrounding tissue.

Fibrosis is a local reactive response to tissue growth in both endometriosis [3] and cancer [4] while it is secondary to inflammation in inflammatory bowel diseases (IBD) (i.e., Chron’s disease) [5]. A correct diagnosis is of key importance, as treatment varies from the need for bowel segmental resection in case of cancer, to possible bowel surgery in endometriosis cases, to medical anti-inflammatory treatment and endoscopic balloon dilation in IBD. As we discussed in a previous article of ours [6], colon cancer growths typically extend outward from the mucosa and reach the serosa, whereas endometriosis lesions grow inward starting from the serosa, so the differential diagnosis is usually not difficult [2]. Patients with IBD commonly develop bowel strictures that may resemble deep infiltrating endometriosis of the bowel at ultrasound. 

**Figure 1:**
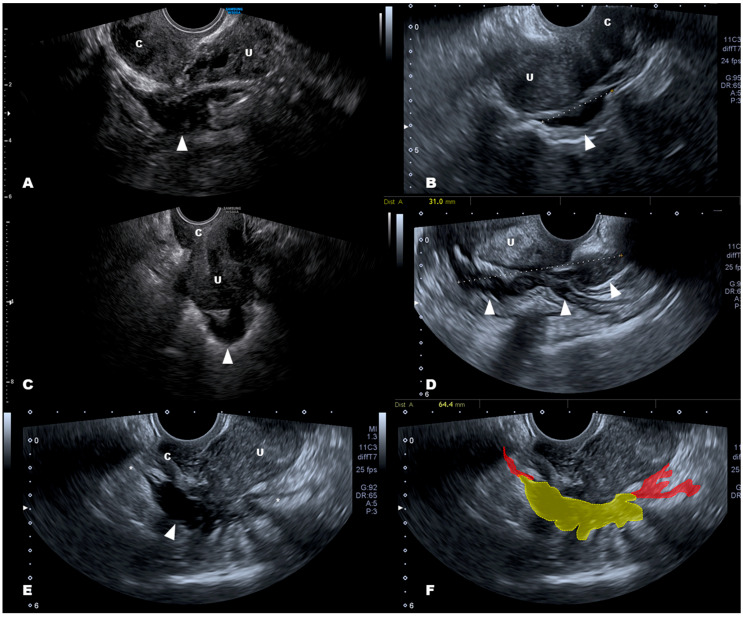
Deep infiltrating endometriosis of the colorectum. (**A**,**B**) show, respectively, typical nodules (arrow) of the medial and the proximal part of the rectum. (**C**) shows an endometriosis nodule of the sigmoid colon. (**D**) shows a large nodule that involves the rectum, from proximal to distal, and the distal sigmoid. (**E**) shows a nodule that involves the medial and proximal part of the rectum (arrow) with long fibrotic tails (*); (**F**) is explains (**E**) where the nodule is in yellow and the fibrotic tails are in red. Abbreviations: U uterus; C cervix.

We agree with Szabó et al. [1] that ESG examination may be of help in the differential diagnosis, as a stiff nodule is not found in IBD. Nevertheless, some aspects related to the technique and diseases should be considered. The strain measurements may show an increased interobserver variability due to the force applied during the transvaginal elastography, even though modern specific ultrasound software provides some information about the pressure made with the probe. The distance between the probe and the lesion represents another limitation, as satisfactory images are more easily acquired if the lesion is relatively close to the probe [7,8,9]. Morphologic and elastographic scores may differ significantly when the bowel lesion is farther away, such as seen in sigmoid endometriosis (Figure 1B,C). Certainly, the majority of endometriosis lesions of the bowel involve the rectum and the rectosigmoid junction [10] or they are quite proximal to the posterior vaginal fornix where the probe is inserted (Figure 1A). Another aspect that should be considered is the case of large nodules that involve the rectum for more than 5 cm (Figure 1D). ESG is based on differences in stiffness induced by the pathological lesion and the normal adjacent tissue that, in cases of large nodules, may be farther away from the probe, so measurements may be less accurate. Similarly, the reactive fibrosis may present as long tails before and after the nodule that may make it difficult to acquire the reference (normal tissue) for stiffness calculation too far away from the probe (Figure 1E,F).

In view of this, further studies are required to assess the potential of ESG in improving the detection rate, potential for differential diagnosis, and intra- and inter-observer reproducibility coefficients of this technique.

## Data Availability

Not applicable.
